# Using SSR-HRM to Identify Closely Related Species in Herbal Medicine Products: A Case Study on Licorice

**DOI:** 10.3389/fphar.2018.00407

**Published:** 2018-04-24

**Authors:** Jingjian Li, Chao Xiong, Xia He, Zhaocen Lu, Xin Zhang, Xiaoyang Chen, Wei Sun

**Affiliations:** ^1^College of Forestry and Landscape Architecture, South China Agricultural University, Guangzhou, China; ^2^College of Pharmacy, Hubei University of Chinese Medicine, Wuhan, China; ^3^Guangxi Institute of Botany, The Chinese Academy of Sciences, Guilin, China; ^4^College of Biological Science and Engineering, Beifang University of Nationalities, Yinchuan, China; ^5^Institute of Chinese Materia Medica, China Academy of Chinese Medical Sciences, Beijing, China

**Keywords:** licorice, SSR markers, high resolution melting, herbal products, species identification

## Abstract

Traditional herbal medicines have played important roles in the ways of life of people around the world since ancient times. Despite the advanced medical technology of the modern world, herbal medicines are still used as popular alternatives to synthetic drugs. Due to the increasing demand for herbal medicines, plant species identification has become an important tool to prevent substitution and adulteration. Here we propose a method for biological assessment of the quality of prescribed species in the Chinese Pharmacopoeia by use of high resolution melting (HRM) analysis of microsatellite loci. We tested this method on licorice, a traditional herbal medicine with a long history. Results showed that nine simple sequence repeat (SSR) markers produced distinct melting curve profiles for the five licorice species investigated using HRM analysis. These results were validated by capillary electrophoresis. We applied this protocol to commercially available licorice products, thus enabling the consistent identification of 11 labels with non-declared *Glycyrrhiza* species. This novel strategy may thus facilitate DNA barcoding as a method of identification of closely related species in herbal medicine products. Based on this study, a brief operating procedure for using the SSR-HRM protocol for herbal authentication is provided.

## Introduction

**FIGURE 1:**
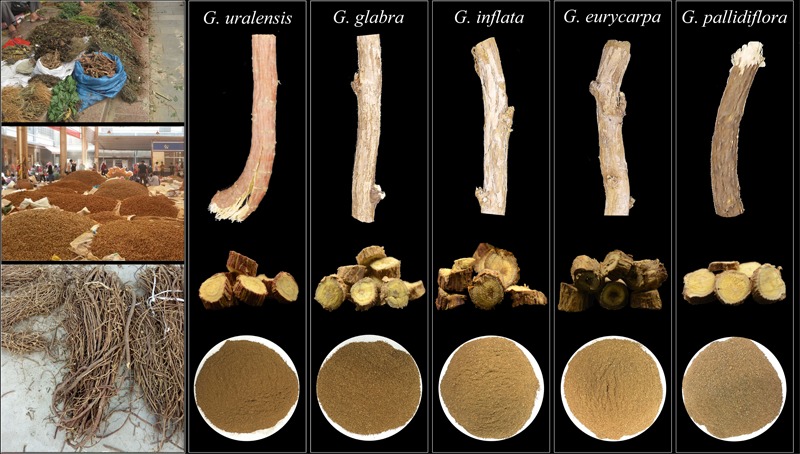
Traditional stall selling herbal medicine products in China.

Recently, both the public and the media have paid a great deal of attention to the efficacy of natural remedies and medicines in preventing chronic disease and improving health. As a result, the trade in raw herbal drugs has increased dramatically throughout the word. According to statistics from the World Health Organization (WHO), ∼80% of the population in developing countries depends on plant-based medical systems to meet their primary healthcare needs^1^. Even in developed countries, raw herbal products are popular substitutes for synthetic drugs. A report from Global Industry Analysts, Inc. predicts that the annual global sales of herbal medicines will have reached US$107 bn by the year 2017 (GIA, 2013). Owing to rapid global industrialization and modernization, diverse medicinal plant products are available in a global market. The Internet also is an important part of this marketplace, since patients can purchase some controlled herbal medicines without a prescription. However, increased accessibility and popularity of herbal medicines has been accompanied by an increase in unscrupulous commercial practices. Manufacturers often incorrectly label herbal products or add cheap or low-quality adulterants to expensive herbal medicines to increase profit. Consequently, suspect or counterfeit herbal products are quite common (Newmaster et al., 2013; Xin et al., 2015). In the United States herbal/dietary supplement market, it is estimated that consumers have a 50% chance of choosing an adulterated product containing an incorrect species (Cordell and Colvard, 2012). China is a biologically resource-rich country, and medicinal plants are one of the fastest-growing segments of the alternative medicine market in China. Due to historical and traditional cultural influences, many unregulated and unregistered stores in China sell freely using traditional stalls (**Figure 1**). Contamination is a persistent problem in this situation. Furthermore, substitution and adulteration of effective herbal ingredients by some other species leads to reductions in the therapeutic effect of the original herbal medicine; this poses a serious risk to end users. In order to prevent herb substitution and admixture in herbal products, the United States Food and Drug Administration regulates labeling of herbal medicines. Manufacturers and distributors are forbidden from selling adulterated or wrongly labeled herbal products, and they are responsible for meeting safety and identification requirements before herbal medicines are made available on the market (The United States Food and Drug Administration, 2014). In addition to the United States, the governments of some European and Asian countries (e.g., the United Kingdom, China, Japan, and Thailand) have also taken steps to strengthen the regulatory oversight of natural remedies and medicines, to ensure that consumers receive accurate information regarding the quality of herbal medicines (World Health Organization [WHO], 2013).

Several methods are used to identify plant species used in herbal medicines. Traditional techniques such as organoleptic, macro-, and microscopic methods have been used to identify herbal medicines since the inception of pharmacognosy (Franz et al., 2007; Li M. et al., 2011; Shen et al., 2014). Despite these methods, the raw materials used to manufacture botanical dietary supplement are typically processed into many forms—including tablets, bulk powder, capsules, and teabags, among others—and processing hampers morphological authentication. In the last decade, herbal medicine identification has begun to employ newer techniques. These include chemical profiling using TLC, HPLC-UV, GC, and ^1^H NMR spectroscopy; such techniques are commonly used for major secondary metabolite detection (Lau et al., 2012; Wu et al., 2015; Liu et al., 2017). However, chemical profiles change with age, differences in physiological condition, and length and type of storage. Thus, it can still be difficult to reliably identify the original plant species present in an herbal medicine.

Identification at the DNA level is more reliable, since DNA is a stable macromolecule that exists in all tissues and DNA sequences are not affected by external factors. Advanced molecular techniques enable researchers to use DNA to analyze samples of many different origins. To date techniques such as amplified fragment length polymorphism (Shaw et al., 2009), sequence characterized amplified regions (Theerakulpisut et al., 2008; Kiran et al., 2010), DNA barcoding (Chen S. et al., 2010; Pang et al., 2013), loop-mediated isothermal amplification (Li et al., 2016b; Zhao et al., 2016), and high resolution melting (HRM) (Li et al., 2016a; Osathanunkul et al., 2016; Sun et al., 2017) have been widely used to identify medicinal plants. Of these techniques, DNA barcoding offers fast, sensitive, and accurate identification of plant species in a wide variety of herbal products. This makes the classification of herbal material simple, and promises to improve the taxonomic accuracy of herbal medicine identification. However, plant identification by DNA barcoding has important limitations. For example, distinguishing between closely related species or sub-species may be difficult due to the fact that the limited number of markers used in DNA barcoding has limited discrimination capability (Li D.Z. et al., 2011). Therefore, a complementary method should be used when authenticating closely related species.

Licorice is a very common species used in Chinese Herbal Medicine and Japanese Kampo Medicine. Pharmacologically, licorice has anti-mutagenic, anticancer, antioxidative, antidiabetic, and antiviral activities and is commonly used in primary health care (Tang et al., 2015). Phytochemical experiments have demonstrated that flavonoids and triterpene saponins are the main bioactive compounds present in licorice. The various flavonoids in licorice possess a range of pharmacological activities (Sumner et al., 2015; Ji et al., 2016), while glycyrrhizin is the main triterpene saponin present in licorice and can be used as a natural sweetener. Licorice is a valuable commodity traded throughout the world, both as herbal/dietary supplement and as a phytochemical extract (Simmler et al., 2015). In the Chinese Pharmacopoeia, three species are described as licorice: *Glycyrrhiza uralensis, Glycyrrhiza glabra*, and *Glycyrrhiza inflata* (**Figure 1**). In Chinese Gan-Cao and Japanese Kampo Medicine, these three species are considered to be equivalent and are used without distinction (National Pharmacopoeia Committee, 2010; Japanese Pharmacopoeia Editorial Committee, 2011). Other traditional or herbal medicines can also consist of multiple species, such as Akebiae Caulis (*Akebia quinata, Akebia trifoliata* var. australis, or *Akebia trifoliata*), Rhei Radix et Rhizoma (*Rheum palmatum, Rheum tanguticum*, or *Rheum officinale*), Epimedii Folium (*Epimedium sagittatum, Epimedium pubescens, Epimedium brevicornum*, or *Epimedium koreanum*), *Lonicera japonica* (*Lonicera fulvotomentosa, Lonicera macranthoides, Lonicera confusa*, or *Lonicera hypoglauca*) (World Health Organization [WHO], 1999; National Pharmacopoeia Committee, 2010). Just to name a few here. In the past few years, the rapidly growing demand for raw material has endangered wild licorice species (Huang et al., 2010), and therefore substitutions have appeared. According to previous reports, the plant materials most frequently used to adulterate licorice are the closely related species *Glycyrrhiza pallidiflora* and *Glycyrrhiza eurycarpa* (Ayiguli et al., 2007; Ma et al., 2016). Prior research has shown that the roots of these licorice species exhibit marked differences in metabolite abundance. Species differ in terms of the abundance of flavanones, chalcones, and other phenolic constituents, and may therefore have different therapeutic efficacy (Farag et al., 2012). Licorice species have been identified by root morphology, component properties, and DNA barcoding, but all did not evolve satisfactorily (Kondo et al., 2007; Simmler et al., 2015; Yang et al., 2017).

In the past two years, our team has integrated plant DNA barcoding and HRM analysis to authenticate medicinal plant species and quantify the concentrations of adulterants in commercial herbal products (Song et al., 2016; Xiong et al., 2016). In this paper, we examine microsatellite (simple sequence repeat, SSR) loci genotypes using HRM (SSR-HRM) to identify closely related species of *Glycyrrhiza*. SSR-HRM has recently been used for both taxonomic identification and the detection of adulterants in food and crop products. Here we appraise whether this technique can be equally applied to species discrimination in constituents of herbal products. This work offers a reliable and robust set of SSR markers for licorice species identification, and also provides a reference for the quality control and management strategies that can be used to protect the integrity of other herbal products.

## Materials and Methods

### Plant Materials and DNA Isolation

To develop an effective SSR-HRM assay, in this study we surveyed, documented, collected, and selected 161 specimens of original licorice plants as well as common adulterants (Supplementary Table S1). Leaf material was collected from these plants and was desiccated *in silica* gel before DNA extraction. To verify the effectiveness of the SSR-HRM method in detecting adulteration of commercially available licorice products, 47 products labeled as *G. uralensis* and/or “gan cao” in Chinese were purchased from different raw herbal material retail markets or online stores. Total DNA was extracted according to a modified CTAB protocol as reported by Sun et al. (2016). High-quality genomic DNA is an important prerequisite for accurate HRM-based plant identification. Generally, the concentrations of polysaccharide and polyphenol compounds in root and rhizome-based herbs are relatively high. These interferents must be removed using polyvinylpyrrolidone during the early stages of DNA extraction. In addition, licorice roots are also rich in fiber, and so the volume of material and of the corresponding extraction solution should be increased. DNA concentration was quantified using a spectrophotometer (Qubit 3.0, Invitrogen Co., United States) and was then adjusted to a 100 ng/μL working concentration and stored at -20°C until needed.

### SSR Primers Mining and Screening

Genome data for *G. uralensis* was retrieved from the ngs-data-archive^2^. To identify potential SSR markers, the *G. uralensis* genome sequences were screened using MISA^3^. We searched for loci with di- and trinucleotide motifs of at least eight or seven repeats, respectively, or with five repeat units for tetra-, penta-, and hexanucleotide SSRs. After screening, more than 100,000 putative microsatellite loci were identified. Because so many microsatellite loci were found in the genome, we selected at random 350 putative microsatellite loci distributed in different scaffolds and in different locations to design SSR primers.

Microsatellite loci were initially amplified using five DNA samples from the five licorice species. PCR was carried out in a final volume of 20 μL containing: 10 μL of 2× Taq PCR mix (Sangon Biotech Co., Ltd., China), 1 μL of DNA template (100 ng/μL), 1 μL of each primer (10 μM), and 7 μL of ultrapure water. The following PCR program was used: 94°C for 3 min; 35 cycles at 94°C for 30 s, at primer annealing temperature for 30 s, and at 72°C for 30 s; and a final extension cycle at 72°C for 7 min. PCR products were separated using 8% non-denaturing polyacrylamide gels. SSR markers showing polymorphism among the five species examined were chosen to amplify in 161 samples. Only those SSR markers that were monomorphic within each species were used for further SSR-HRM analysis (**Table 1**).

**Table 1:** Characteristics of the nine novel SSR primers.

SSR name	Repeat motif	Sequence (5′ → 3′)	Temperature	(°C)	Expected size (bp)
GM-S1	(AT)^8^(GT)^10^	CAGCCTCAGGACCTTCTACC	55	∼190
		CTTCTCTTCACCGAAACCAC		
GM-S2	(AG)^14^	GGCAACTCTAGCGTTTTCTC	55	∼300
		GTGTAATCACAGCAGGGAAGT		
GM-S3	(AG)^7^(TG)^9^	GCACTAGCACACGATTTGAT	53	∼340
		CCCTCCTCCTTTCTTACTTC		
GM-S4	(CCA)^5^N^18^(CT)^8^	AGTGATTTAGGGCTGAGGTG	55	∼370
		GCCAGTGTGGATGAAAGATG		
GM-S5	(CA)^13^	GTGAACCGTCTCGTCCCTC	53	∼330
		TCCGCTTCCCGATAATGTT		
GM-S6	(AAG)^6^(AAC)^8^N^52^(AT)^9^	CTATCACTGCACAGTGGCTT	53	∼300
		ACACCCTTCTCCTTCAAATG		
GM-S7	(GAA)^8^	CCTTCCTTTGATTTCACAGC	53	∼300
		ACATCTCTCTCCACGCAGC		
GM-S8	(CGGAGA)^5^N^44^(CT)^9^	AGGAAAAGGGAAAGGCAAGC	53	∼260
		AAGAGCGTTCGTTGGCAGAG		
GM-S9	(CATC)^7^N^13^(TAA)^6^	CCACTGTCCACTCCCATTTA	55	∼300
		GTTAGTTCAGGCGTGCGTA		

### HRM-PCR Amplification and Data Analysis

SSR-HRM analysis was performed using a Rotor-Gene Q MDx (QIAGEN) real-time PCR machine. The HRM reaction mixture contained 50 ng of genomic DNA, 5 μL of 2× HRM master mix (QIAGEN, Germany), 0.5 μL of 10 μmol/L of both forward and reverse primers, and distilled water was added to reach the final volume. HRM-PCR was conducted in a 72-well carousel with the following reaction conditions: an initial denaturing step of 94°C for 3 min, followed by 40 cycles of 94°C for 30 s, primer annealing temperature for 30 s, and 72°C for 30 s. Fluorescence data was collected at the end of each extension step of each PCR cycle. HRM was performed immediately after PCR amplification. For HRM, the temperature rose from 70 to 90°C at 0.15°C increments with a 2 s hold time for each acquisition step. As the temperature increased, fluorescence was continuously monitored. A sharp drop in fluorescence occurred as the sample temperature reached the denaturation temperature. Data was analyzed using the Rotor-Gene Q software. Melting curve profiles were processed in three steps: (1) normalization, (2) temperature shift, and (3) difference plot visualization. Melt data were first normalized by removing background fluorescence and adjusting the fluorescence signals for the variation among wells. The negative derivative of the fluorescence (*F*) over temperature (*T*) (d*F*/d*T*) curve identifies the melt temperature (*T*_m_), and the normalized raw curve shows the decrease in fluorescence against increasing temperature. Finally, to create more easily distinguishable plots, a base melting curve was selected. To genotype commercial licorice products, a two-step procedure was used to assess the similarity between unknown HRM curves (i.e., commercial samples) and known ones (i.e., original plants). First, the normalized HRM curve of the commercial product was compared to known normalized HRM profiles of the five *Glycyrrhiza* species. The HRM profile of the unknown genotype either resembled one of the known samples or generated a unique melting curve. Second, the HRM genotype confidence percentage (GCP) of the licorice product was calculated by setting each known species as a “genotype” (i.e., reference species), and the confidence threshold was set to 90%.

### Capillary Electrophoresis

To validate the results of HRM analysis, we labeled all forward SSR primers with the fluorescent dye 6-FAM and performed PCR before conducting capillary electrophoresis. The PCR was performed in a 10 μL reaction mixture consisting of 0.4 μL of genomic DNA, 1 μL of 10× buffer, 0.3 μL of 25 mmol/L MgCl_2_, 0.2 μL of 10 mmol/L dNTPs, 0.1 μL each of 10 μm/L forward and reverse primers, 1 μL of 10 mg/mL BSA-V, 0.1 μL of 5 U/L Taq polymerase, and distilled water up to the final volume. PCR amplification reaction parameters were as previously mentioned. Capillary electrophoresis test samples were prepared by diluting amplified SSR fragments and dispensing in each well 2 μL of the diluted products and 9 μL Hi-Dye formamide solution premixed with 500 Liz^TM^ dye standard following the manufacturer’s instructions (Life Technologies). Samples were then denatured at 95°C for 3 min followed by quick cooling on ice. Fragment analyzes were conducted on an ABI 3730XL genetic analyzer (Applied Biosystems, Foster City, CA, United States). During capillary electrophoresis, the separation processes were automatically recorded in a single GeneScan file; this file was then analyzed with GeneMapper V4.0 (Applied Biosystems, Foster City, CA, United States).

## Results

### Data Mining and Primer Evaluation

The genome sequence of *G. uralensis* was extracted from a genome data bank for SSR marker mining. In total, more than 10,000 microsatellite loci were identified, of which 350 microsatellite loci with dimer, trimer, tetramer, pentamer, and hexamer motifs with lengths longer than 10 bp were randomly selected for further analysis. Mononucleotide repeats were excluded from this study due to the fact that they are associated with unstable polymorphism. The 350 SSR primer pairs were used to amplify DNA-fragments from five *Glycyrrhiza* species, and the resulting PCR products were analyzed using polyacrylamide gel electrophoresis to define their repeat numbers and to detect polymorphism. The electrophoresis data showed that the discriminatory power of the SSR primers ranged from 0 to 100%, and the SSR markers could be separated into five classes as follows: (1) none of the five *Glycyrrhiza* species could be distinguished from each other, (2) two of five species could be distinguished from one another, (3) three of five species could be distinguished, (4) four of five species could be distinguished, and (5) all five species could be distinguished from one another. After one round of screening, 83 primer pairs that had the ability to distinguish five *Glycyrrhiza* species were qualified for the next step of testing. In this round, the 83 candidates were characterized using 161 samples of the five species. Finally, nine primer pairs which displayed monomorphism within samples of same species were used for SSR-HRM analysis in this study (**Table 1**).

### Microsatellite Genotyping of Five *Glycyrrhiza* Species Using HRM Analysis

**FIGURE 2:**
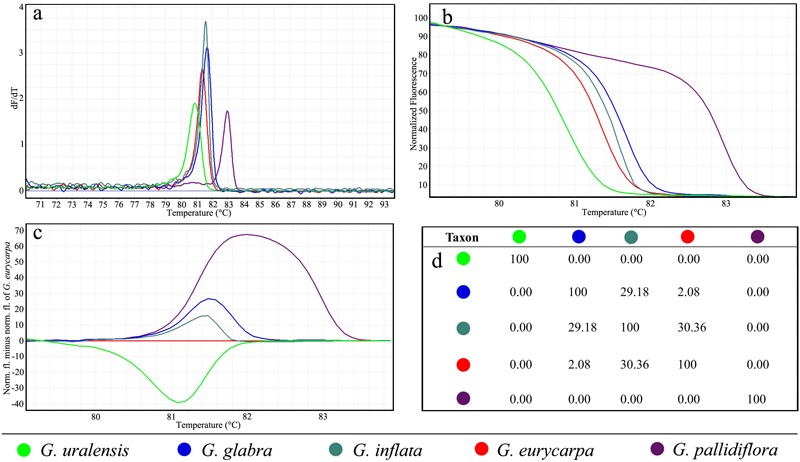
Microsatellite typing of five *Glycyrrhiza* species using HRM analysis with marker GM-S9. **(a)** Derivative melting curves. **(b)** Normalized melting curves. **(c)** Difference plot curves. **(d)** Average genotype confidence percentages.

The potential resolving power of HRM is much greater than conventional melting curve analysis and PAGE gel electrophoresis, since HRM permits distinguishing between the shapes of melting curves from different amplicons even when they have the same *T*_m_. Here we used both *T*_m_ values and the shapes of melting curves to assess differences among five *Glycyrrhiza* species (**Figure 2**). **Figure 2a** presents conventional derivative melting curves, which shows peaks indicating the *T*_m_ values for the GM-S9 SSR fragment amplified from the five *Glycyrrhiza* species. The melting curve is produced by slowly melting the DNA of tested samples in the presence of a dsDNA binding dye within a range of temperatures. The melting temperature peaks of the tested plant species are calculated as *T*_m_ values (**Table 2**). The *T*_m_ values of *G. inflata*, and *G. glabra* were similar, but these species could be distinguished by plotting normalized melting curves (**Figure 2b**) and difference plot melting curves (**Figure 2c**). Analysis of the normalized melting curves of the SSR marker GM-S9 (**Figure 2b**) proved that all test species could be easily distinguished. *G. uralensis* and *G. pallidiflora* were easily distinguished by their distinct melting curves, while in contrast the curve profiles of *G. inflata* and *G. eurycarpa* were similar and were not easy to visually distinguish from each other. To better visualize small differences between these two species, close examination of difference plot melting curves was performed with the melting curve of *G. eurycarpa* used as the baseline; this revealed that a part of the curve was outside the 90% CI curve, suggesting that the melt curves of *G. inflata* and *G. eurycarpa* are indeed different (**Figure 2c**). Similar results were obtained for all other eight SSR markers except GM-S4; this marker could not be amplified in *G. inflata* (Supplementary Figure S1). Furthermore, the confidence values for the similarity between each pair of the *Glycyrrhiza* species was calculated by setting each species as a “genotype” (reference species) with a confidence threshold that was set in an appropriate control. As shown in **Figure 2d**, the highest GCP (30.36) was found between *G. inflata* and *G. eurycarpa*, while the lowest (0) was between *G. uralensis* and other species. According to calculated GCPs, the most informative microsatellite marker was GM-S1, while GM-S7 was the least informative. However, while GM-S1 was the best marker for this study, other SSR primers—either alone or in combination—might be better able to identify other *Glycyrrhiza* species.

**Table 2:** Results of *T*_m_ values achieved by SSR-HRM analysis from control genomic DNA of five *Glycyrrhiza* species.

SSR primers	Melting curve *T*_m_ values (°C) ± SD				
	*G. uralensis*	*G. glabra*	*G. inflate*	*G. eurycarpa*	*G. pallidiflora*
GM-S1	75.42 ± 0.05	75.23 ± 0.12	75.67 ± 0.05	76.08 ± 0.08	76.04 ± 0.05
GM-S2	78.81 ± 0.08	78.53 ± 0.10	78.11 ± 0.05	78.15 ± 0.06	79.42 ± 0.05
GM-S3	83.13 ± 0.05	82.95 ± 0.07	83.25 ± 0.05	83.29 ± 0.06	83.40 ± 0.04
GM-S4	81.70 ± 0.06	80.18 ± 0.10	/	80.84 ± 0.05	81.61 ± 0.05
GM-S5	80.55 ± 0.10	80.36 ± 0.07	80.73 ± 0.06	80.67 ± 0.06	80.63 ± 0.05
GM-S6	78.67 ± 0.04	78.91 ± 0.08	78.64 ± 0.04	78.69 ± 0.04	76.49 ± 0.08
GM-S7	80.23 ± 0.05	81.01 ± 0.06	81.14 ± 0.05	81.10 ± 0.06	79.18 ± 0.07
GM-S8	79.87 ± 0.10	79.59 ± 0.10	80.46 ± 0.10	80.54 ± 0.06	79.15 ± 0.08
GM-S9	80.89 ± 0.06	81.66 ± 0.08	81.51 ± 0.04	81.37 ± 0.08	82.97 ± 0.05

### Validation of HRM Results Using Capillary Electrophoresis and DNA Sequencing

**FIGURE 3:**
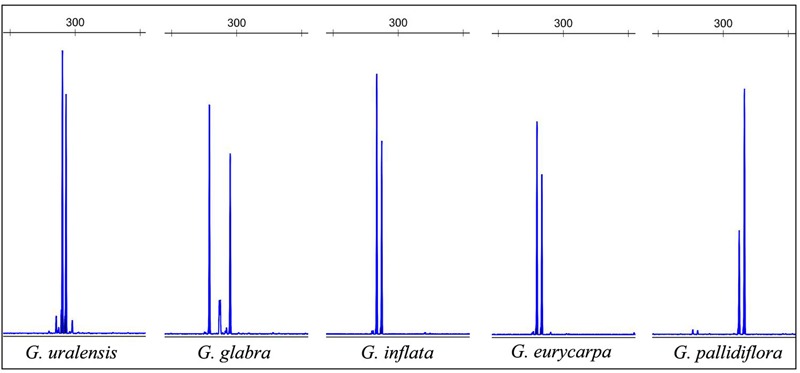
Capillary electrophoresis profiles of the genotypes analyzed with marker GM-S9.

To validate the HRM results, we performed capillary electrophoresis on heat denatured PCR products labeled with FAM. These results revealed that each *Glycyrrhiza* species had distinct patterns and that the five species could be differentiated from each other. This demonstrates that the newly designed SSR primers can be used for licorice species identification (**Figure 3** and Supplementary Figure S2) and is consistent with our HRM results. Interestingly, analysis of sequenced DNA found that some amplicons not only exhibited different microsatellite alleles, but also contained single nucleotide polymorphisms (SNPs) in regions flanking the SSR (data not shown here due to the poor quality of SSR sequencing). The presence of SNPs in these flanking sequences has been previously reported in other species (Distefano et al., 2012). HRM is a powerful method that can be used to detect SNPs, which means that relative to capillary electrophoresis, its higher sensitivity means that it is better able to discriminate PCR products of the same size that have different base compositions.

### Survey of Commercial Samples

**FIGURE 4:**
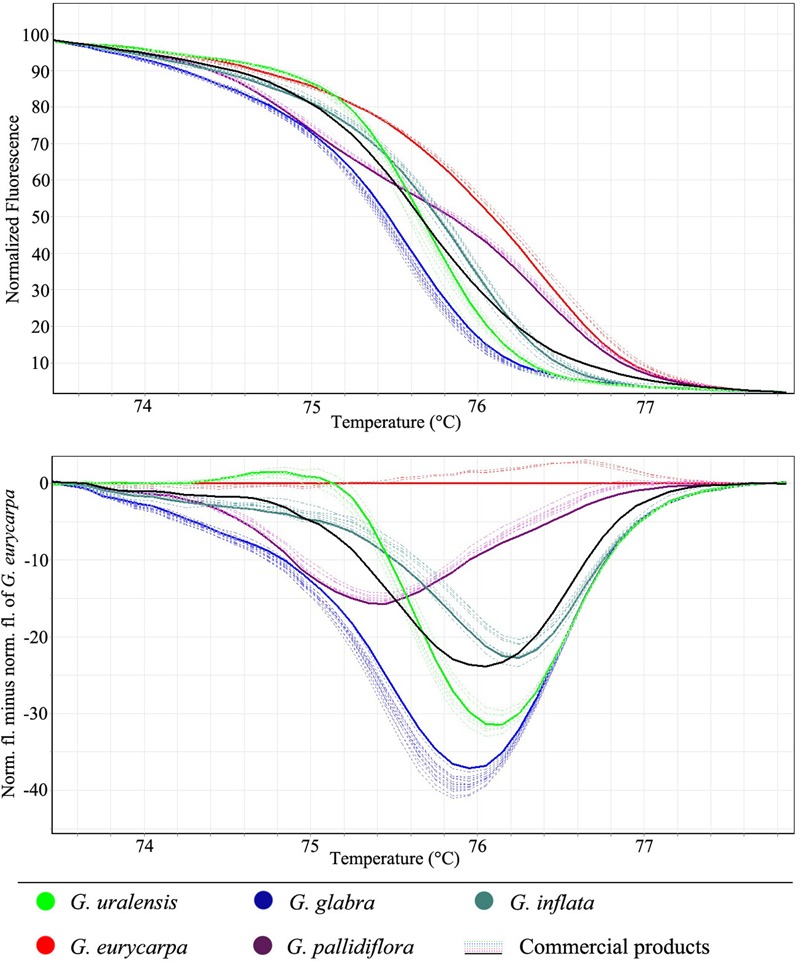
Microsatellite typing of the commercial licorice products using HRM with marker GM-S1.

After the confirmation that each species could be identified using the SSR-HRM protocol, we used the same method to identify the species used in commercial licorice products. A total of 47 test samples were collected from products sourced from both pharmacies and online vendors. All samples were labeled as “gan cao” and were available to consumers and patients. Genomic DNA was extracted from all 47 samples using a modified CTAB method (Sun et al., 2016). DNA samples from the five known *Glycyrrhiza* species were used as controls. The normalized and difference plot curves of the amplicons from the five *Glycyrrhiza* species and 47 commercial licorice products, based on HRM analysis of SSR marker GM-S1 are shown in **Figure 4**. By using each known species as a “genotype” (i.e., reference species), and with the confidence threshold set to 90%, we were able to calculate the degree of confidence in the similarity of each commercial product to each of the five known species (shown in Supplementary Table S2). For example, HRM analysis allowed us to identify *G. uralensis* in sample 13 with a confidence level of 99.6%, which agrees with the labeled information indicating that this product is 100% *G. uralensis* (Supplementary Table S2). However, in sample 4, HRM analysis detected *G. pallidiflora* with a confidence level of 98.9%, which suggested that this product contained an adulterant. The same results were also observed for nine other samples in which *G. pallidiflora* and *G. eurycarpa* were identified with a high level of confidence. It was also noteworthy that the melting curve of tested sample 40 was intermediate between the curves of *G. uralensis* and *G. eurycarpa*; this may indicate that this product is an admixture of *G. uralensis* and *G. eurycarpa* (**Figure 4**). To back up this speculation, TA-cloning was conducted on PCR product from this sample using the universal ITS2 primer. Sequencing of the cloned products confirmed that DNA from two different species were present. We also found that 15 samples (31.9%) were authenticated as *G. glabra*, 11 (23.4%) were authenticated as *G. uralensis*, and 10 (21.3%) were authenticated as *G. inflata*. This confirms that the most common species found in available commercial licorice products are species included in the Chinese Pharmacopoeia. This finding also provides evidence that some adulterants are present in herbal products sold in Chinese markets, and that this adulteration may be a serious issue for the herbal drug industry.

### A Brief Protocol for the Accurate Identification of Closely Related Species in Herbal Medicines Using SSR-HRM

**FIGURE 5:**
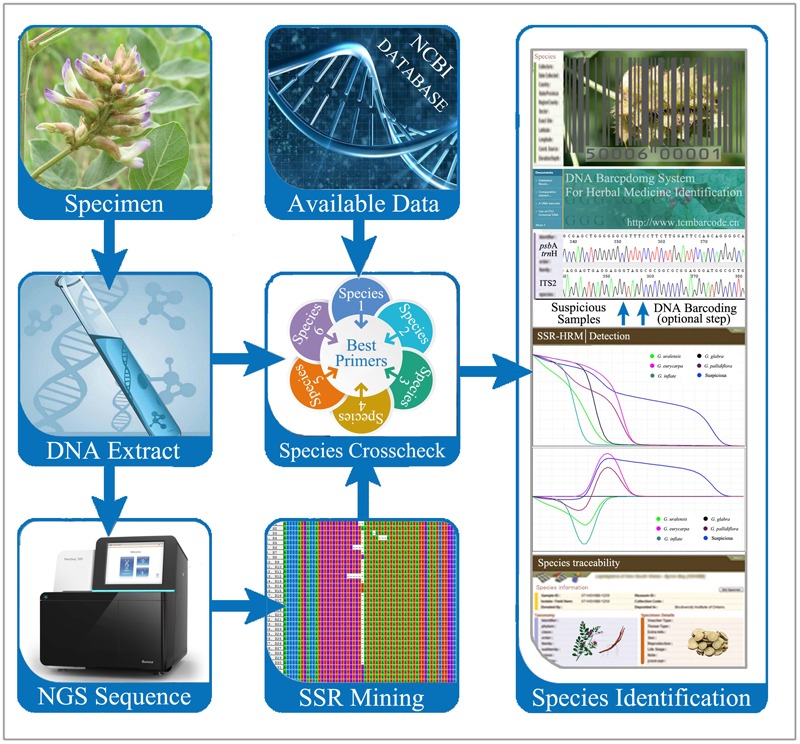
A brief operating procedure for herb authentication using SSR-HRM.

The HRM method has been widely used for many purposes (Druml and Cichna-Markl, 2014), including the detection of infectious diseases, testing of bacterial pathogens in foods, and the identification of genetically modified organisms. HRM has notable advantages due to its simplicity, rapidity, sensitivity, and accuracy. Based on the protocol used in this study, we propose a brief operating procedure for traditional herbal medicine authentication using SSR-HRM (**Figure 5**). First, genomic DNA must be extracted from both the authentic medicinal plant and the adulterant(s). The extracted DNA can then be used to acquire a microsatellite library using next-generation sequencing. Second, the fragments of the selected microsatellite loci should be amplified by PCR and the amplicons run on a PAGE gel to assess polymorphism. Third, SSR-HRM amplification is performed using primers for polymorphic loci with DNA from both authentic, known plants as well as their adulterants. The best primer pair—i.e., those that are both stable and efficient—are then rechecked. Fourth, a preliminary test is conducted to detect adulterants in commercial products by performing SSR-HRM PCR using the selected primers. Fifth, if the SSR primers do not amplify in the adulterant samples, DNA barcoding can be used to assist with species identification.

## Discussion

### Herbal Medicine Supply and Its Safety Issues Around the World

The supply and safety of herbal medicines are major global public health issues, and are particularly important in heavily populated countries such as China and India. Rapid modernization and globalization continue to have a profound impact on both herbal medicine supply and safety. Many traditional herbal medicine products are available not only in pharmacies but are now also sold on the Internet. According to a WHO survey, in 99 out of 142 countries a majority of herbal products can be purchased without prescription, and many natural remedies and medicines are used for self-medication, and/or are bought or prepared by friends, acquaintances, or the patient (World Health Organization [WHO], 2004). These trends bring highlight the importance of the quality and safety of the products used. Moreover, with increased use of traditional herbal medicines, reports of adverse reactions have also increased.

In China, a country where traditional therapies are widely used to complement conventional medicine, there were 9854 known reported cases of adverse drug reactions in 2002 alone, which was higher than the 4000 that occurred between 1990 and 1999 (Li et al., 2016b). Numerous and irrefutable cases of adverse herbal drug reactions have been reported in the literature (Zhou et al., 2004; Chen W.L. et al., 2010; Liu et al., 2011; Dunnick and Nyska, 2013; Kim et al., 2013). Misidentification of medicinal plants and mislabeling herbal medicinal products can sometimes cause adverse consequences, so it is of great importance to examine herbal medicines for the presence of adulterants. The Former Director General of the WHO Dr. Lee Jong-wook said that “WHO supports traditional and alternative medicines when these have demonstrated benefits for the patient and minimal risks.” Thus, it is easy to conclude that WHO highlighted the contribution of traditional medicine in improving health care. “But as more people use these medicines, governments should have the tools to ensure all stakeholders have the best information about their benefits and their risks,” Lee added. Whether intentional or not, substitution and adulterants are highly undesirable.

To address this issue, DNA barcoding technology provides a novel method that can be used to confirm the identification of raw herbal materials and to develop a standard quality within the marketplace. This technology uses a short fragment (<1000 bp) of the genome (also called a “DNA barcode”) to identify species (Lahaye et al., 2008). In plants, however, this method cannot be used to distinguish between in closely related species or sub-species, due to the slow evolution and limited divergence in these barcodes. Further studies are therefore necessary for this protocol improvement, especially exploiting a complementary tool to facilitate DNA barcoding for a broader coverage of plant species. Here we argue that an SSR-based method may serve as a useful alternative. SSR markers result from differences in the lengths of mono- to hexa-nucleotide repeat sequences. These markers are popular because of their high stability and co-dominant transmission. Moreover, conserved microsatellites can be readily used for taxonomic studies (Küpper et al., 2008; Tuler et al., 2015). Because of these advantages, the SSR method can be effective and for the identification of closely related species, and can therefore assist in identifying ingredients present in herbal medicine.

### The Reliability of SSR-HRM for *Glycyrrhiza* Herbal Supplement Species Identification

This study is the first to use SSR-HRM to detect the species composition in currently available licorice herbal supplements. Our results indicated that genomic DNA can be obtained from medicinal parts (i.e., dried roots) as well as from their processed or modified forms (i.e., decoction pieces or powder), and its quality was suitable for HRM analysis. We used 161 voucher samples to verify the reliability of the newly developed SSR markers. SSR patterns obtained from polyacrylamide gel and HRM analyzes were similar, which indicated that these SSR markers were able to accurately distinguish between the five species. Therefore, we speculate that the SSR markers developed in this study have the power to identify the original plant species used in commercial *Glycyrrhiza* herbal supplements as well as their substitutes.

Among the 47 herbal samples sold in pharmacies and Internet in China as “gan cao,” we confirmed that 75% of the samples were authentic, while the rest of the samples included adulterants. Seven test samples were authenticated as *G. pallidiflora*, and three of the remaining samples matched *G. eurycarpa*. One sample was authenticated as an admixture of *G. uralensis* and *G. eurycarpa*. This result demonstrates that licorice products available in the market may have complex sources, and that samples purchased on the Internet may contain adulterants. Using such substitute species as dietary supplements may be detrimental to patients and consumers and may ultimately also be harmful to public health. Although 36 out of 47 (75%) products tested were identified as the labeled species, we speculate that a similar analysis of a larger number of samples from additional markets could lead to figures lower than 75%. Detecting adulteration in herbal medicines has been reported in previous studies, and therefore it is not surprising that licorice produce are also mislabeled. The China Food and Drug Administration should monitor commercial licorice dietary supplements. The SSR-HRM method developed here is an effective way to accurately distinguish between closely related species in herbal products.

### Future Perspectives for SSR-HRM in Herbal Medicine Identification

It has been shown that HRM is a rapid and cost-effective approach for detecting sequence variants in clinical research and diagnostics (Akiyoshi et al., 2013). HRM has also been applied in plants to detect SNPs used for genotype identification (Wu et al., 2008). Potentially, HRM can also be used for the identification of homozygous and (known) heterozygous variants (Montgomery et al., 2010). The time cost and analysis is similar to conventional PCR but HRM does not require post-PCR procedures (e.g., gel electrophoresis). Although some studies have described HRM applications where microsatellite loci are used to identify food adulterants (Ganopoulos et al., 2011; Bosmali et al., 2012), the merits of employing this method have not been fully reported. In this study, we analyzed nine novel SSR markers using HRM and compared the results with those produced from capillary electrophoresis analysis. DNA patterns from five *Glycyrrhiza* species were distinguished successfully using both applications. Although the power of both methods was the same, in general HRM is a more accurate approach and can also detect SNPs in the sequences flanking SSR motifs. Therefore, the use of HRM may be helpful to overcome the limits of capillary electrophoresis-based SSR analyzes. This is especially true in cases where SSR are monomorphic but SNPs are present elsewhere in the amplicon. Nevertheless, SSR-HRM is still not widely used to identify closely related species in the herbal medicine supply chain. In the future, the use of this application for examining the contents of herbal products will likely increase.

The most important properties for routine techniques used to examine the contents of herbal medicines are: the robustness of the process, the availability of testing devices, and the existence of extensive and accessible primer resources. Despite it proven effectiveness, SSR-HRM is still in the initial stage of being adopted as a routine method used to identify the contents of herbal products, and its use for herbal product traceability remains limited due to the lack of SSR primers. In fact, many techniques that are now well-established suffered from a similar limitation early on but have since developed into robust, universal tools (e.g., DNA barcoding). Therefore, there is a need to develop and establish a primer database for SSR-HRM as a universal tool for herbal medicine identification. In last decade, the emergence of next-generation sequencing technologies has increased the availability of molecular markers, including genomic SSRs and SSRs from expressed sequence tags (Wu et al., 2017). Hence, these resources can be used to establish reference SSR databases for herbal medicine identification, and continual updating can guarantee the integrity of the SSR primer database and improve its application. In conclusion, the use of SSR-HRM for the identification of closely related species in herbal products appears to be promising but remains to be fully exploited. In the future, SSR-HRM will not replace classical DNA barcoding but may provide a supplementary tool to facilitate DNA barcoding to identify closely related species in herbal medicine products.

## Author Contributions

JL conceived and designed the experiments. JL, CX, and XH performed the experiments. JL analyzed the data. CX, XZ, and ZL contributed reagents/materials/equipment. JL wrote the paper. XC and WS revised and approved the final version of the paper.

## Conflict of Interest Statement

The authors declare that the research was conducted in the absence of any commercial or financial relationships that could be construed as a potential conflict of interest.

## Footnotes

1 http://www.worldwildlife.org/what/globalmarkets/wildlifetrade/faqs-medicinalplant.html

2 http://ngs-data-archive.psc.riken.jp/Gur-genome/download.pl

3 http://pgrc.ipk-gatersleben.de/misa/
