# Supplementary material for: Using SSR-HRM to Identify Closely Related Species in Herbal Medicine Products: A Case Study on Licorice
Source: Front Pharmacol. 2018 Apr 24;9:407. doi: 10.3389/fphar.2018.00407 (PMC5928318; doi:10.3389/fphar.2018.00407)
Supplement: Supplementary file 1 [file Data_Sheet_1.DOC]

**TITLE:** Using SSR-HRM to identify closely related species in herbal medicine products a case study on licorice

**AUTHORS:** Jingjian Li ^1^**^†^**, Chao Xiong ^2^**^†^**, Xia He ^1^, Zhaocen Lu ^3^, Xin Zhang ^4^, Xiaoyang Chen ^1^ and Wei Sun ^5^

**SUPPLEMENTARY INFORMATION**

**Supplementary Table S1.** Plant materials and commercial products used in this study.

**Supplementary Table S2.** HRM detection of the commercial licorice products using SSR marker GM-S1

**Supplementary Fig. S1.** Microsatellite typing of five *Glycyrrhiza* species using HRM analysis.

**Supplementary Fig. S2.** Capillary electrophoresis profiles of the genotypes analyzed with the newly designed SSR markers.

**Table S1. Plant materials and commercial products used in this study.**

| **Samples** | **Origin** | **Samples from** | **Voucher No.** | **Total** |
| --- | --- | --- | --- | --- |
| *G. uralensis* | Original plant | Gansu, Ningxia, Neimenggu China | GC001GU01~28 | 28 |
| *G. glabra* | Original plant | Gansu, Ningxia, Xinjiang China | GC002GG01~35 | 35 |
| *G. inflata* | Original plant | Gansu, Ningxia China | GC003GI01~31 | 31 |
| *G. eurycarpa* | Original plant | Gansu, Neimenggu China | GC004GE01~31 | 31 |
| *G. pallidiflora* | Original plant | Gansu, Neimenggu China | GC005GP01~36 | 36 |
| *G. uralensis* | Commercial product | Neimenggu, Ningxia, Anhui China | GC006CM01~06 | 6 |
| “Gancao” | Commercial product | Anhui, Sichuan, Gansu, Guangdong, Hubei, Shanxi China | GC006CM07~47 | 41 |

**Table S2.** **HRM detection of the commercial licorice products using SSR marker GM-S1.**

| **Sample No.** | **Claimed *Glycyrrhiza* species** | **HRM detected species** | **Genotype confidence (%)** |
| --- | --- | --- | --- |
| Com-01 | “gancao” | *G*. *glabra* | 91.52 |
| Com-02 | “gancao” | *G*. *glabra* | 92.74 |
| Com-03 | “gancao” | *G*. *glabra* | 96.61 |
| Com-04 | “gancao” | *G*. *pallidiflora* | 98.85 |
| Com-05 | “gancao” | *G*. *glabra* | 99.16 |
| Com-06 | “gancao” | *G*. *inflate* | 98.66 |
| Com-07 | “gancao” | *G*. *inflate* | 98.74 |
| Com-08 | “gancao” | *G*. *pallidiflora* | 93.75 |
| Com-09 | “gancao” | *G*. *eurycarpa* | 94.06 |
| Com-10 | “gancao” | *G*. *uralensis* | 97.05 |
| Com-11 | *G*. *uralensis* | *G*. *pallidiflora* | 96.58 |
| Com-12 | *G*. *uralensis* | *G*. *uralensis* | 98.52 |
| Com-13 | *G*. *uralensis* | *G*. *uralensis* | 99.62 |
| Com-14 | *G*. *uralensis* | *G*. *uralensis* | 95.24 |
| Com-15 | *G*. *uralensis* | *G*. *glabra* | 91.46 |
| Com-16 | *G*. *uralensis* | *G*. *uralensis* | 96.71 |
| Com-17 | “gancao” | *G*. *inflate* | 94.42 |
| Com-18 | “gancao” | *G*. *inflate* | 99.83 |
| Com-19 | “gancao” | *G*. *inflate* | 99.47 |
| Com-20 | “gancao” | *G*. *pallidiflora* | 96.05 |
| Com-21 | “gancao” | *G*. *pallidiflora* | 98.21 |
| Com-22 | “gancao” | *G*. *inflate* | 97.58 |
| Com-23 | “gancao” | *G*. *inflate* | 95.47 |
| Com-24 | “gancao” | *G*. *inflate* | 90.84 |
| Com-25 | “gancao” | *G*. *eurycarpa* | 93.74 |
| Com-26 | “gancao” | *G*. *uralensis* | 96.86 |
| Com-27 | “gancao” | *G*. *pallidiflora* | 97.18 |
| Com-28 | “gancao” | *G*. *eurycarpa* | 98.52 |
| Com-29 | “gancao” | *G*. *uralensis* | 98.1 |
| Com-30 | “gancao” | *G*. *uralensis* | 97.83 |
| Com-31 | “gancao” | *G*. *uralensis* | 93.46 |
| Com-32 | “gancao” | *G*. *glabra* | 91.38 |
| Com-33 | “gancao” | *G*. *glabra* | 93.54 |
| Com-34 | “gancao” | *G*. *inflate* | 97.26 |
| Com-35 | “gancao” | *G*. *glabra* | 96.01 |
| Com-36 | “gancao” | *G*. *glabra* | 96.71 |
| Com-37 | “gancao” | *G*. *glabra* | 92.43 |
| Com-38 | “gancao” | *G*. *pallidiflora* | 93.81 |
| Com-39 | “gancao” | *G*. *inflate* | 90.97 |
| Com-40 | “gancao” | *G*. *eurycarpa* | 64.16 |
| Com-41 | “gancao” | *G*. *glabra* | 96.45 |
| Com-42 | “gancao” | *G*. *uralensis* | 97.49 |
| Com-43 | “gancao” | *G*. *uralensis* | 95.16 |
| Com-44 | “gancao” | *G*. *glabra* | 96.44 |
| Com-45 | “gancao” | *G*. *glabra* | 95.91 |
| Com-46 | “gancao” | *G*. *glabra* | 92.73 |
| Com-47 | “gancao” | *G*. *glabra* | 94.44 |


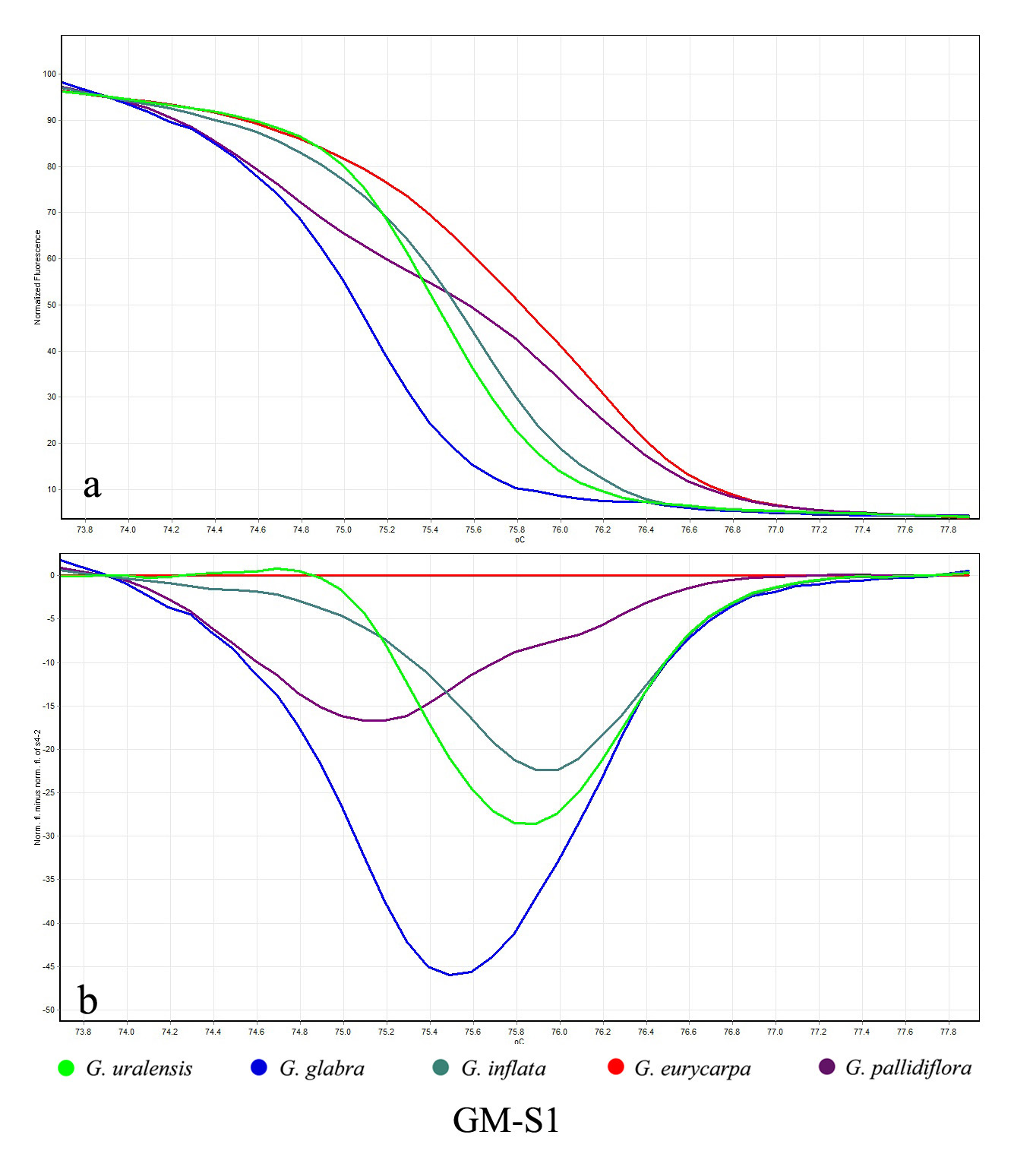

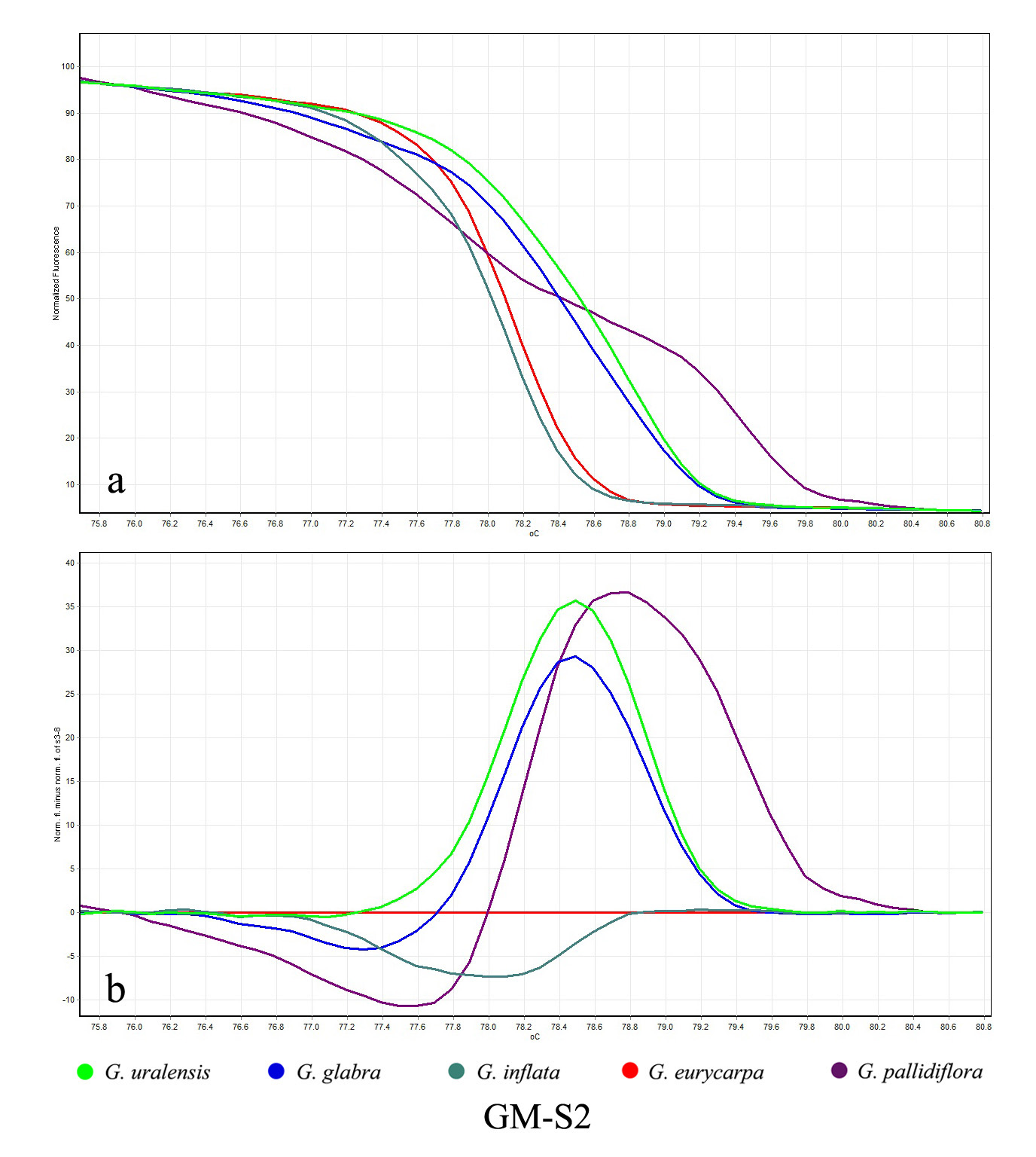

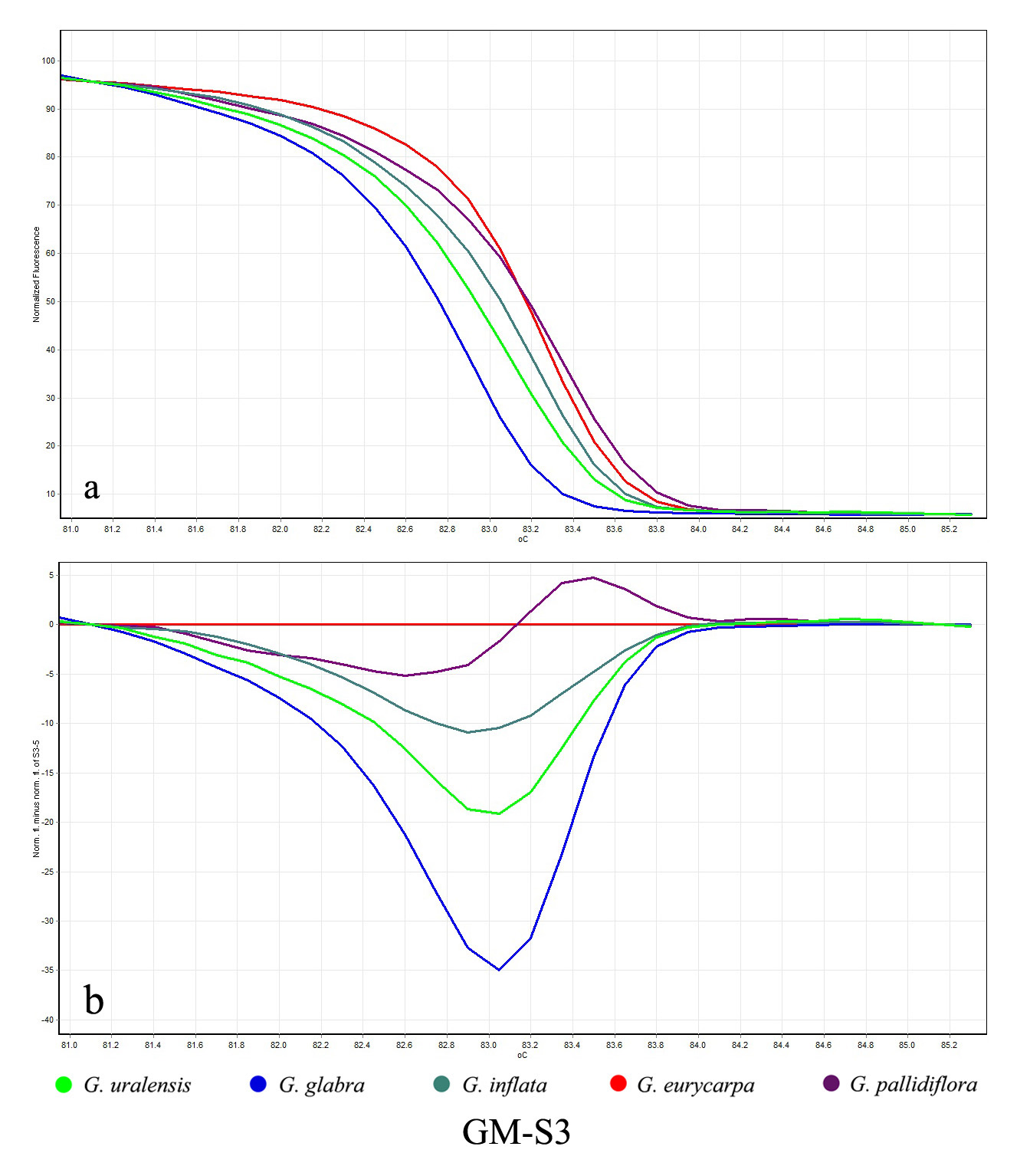

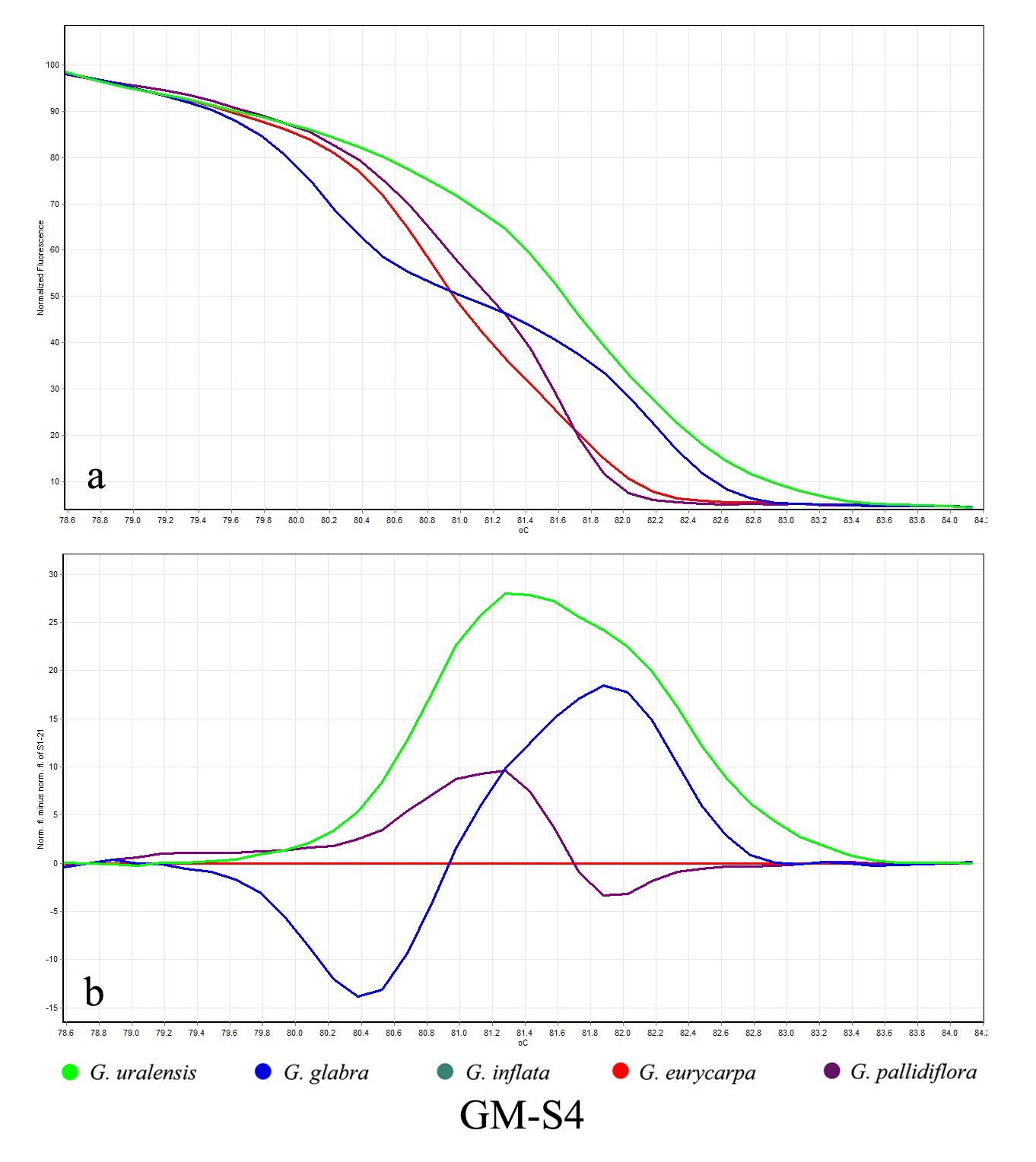

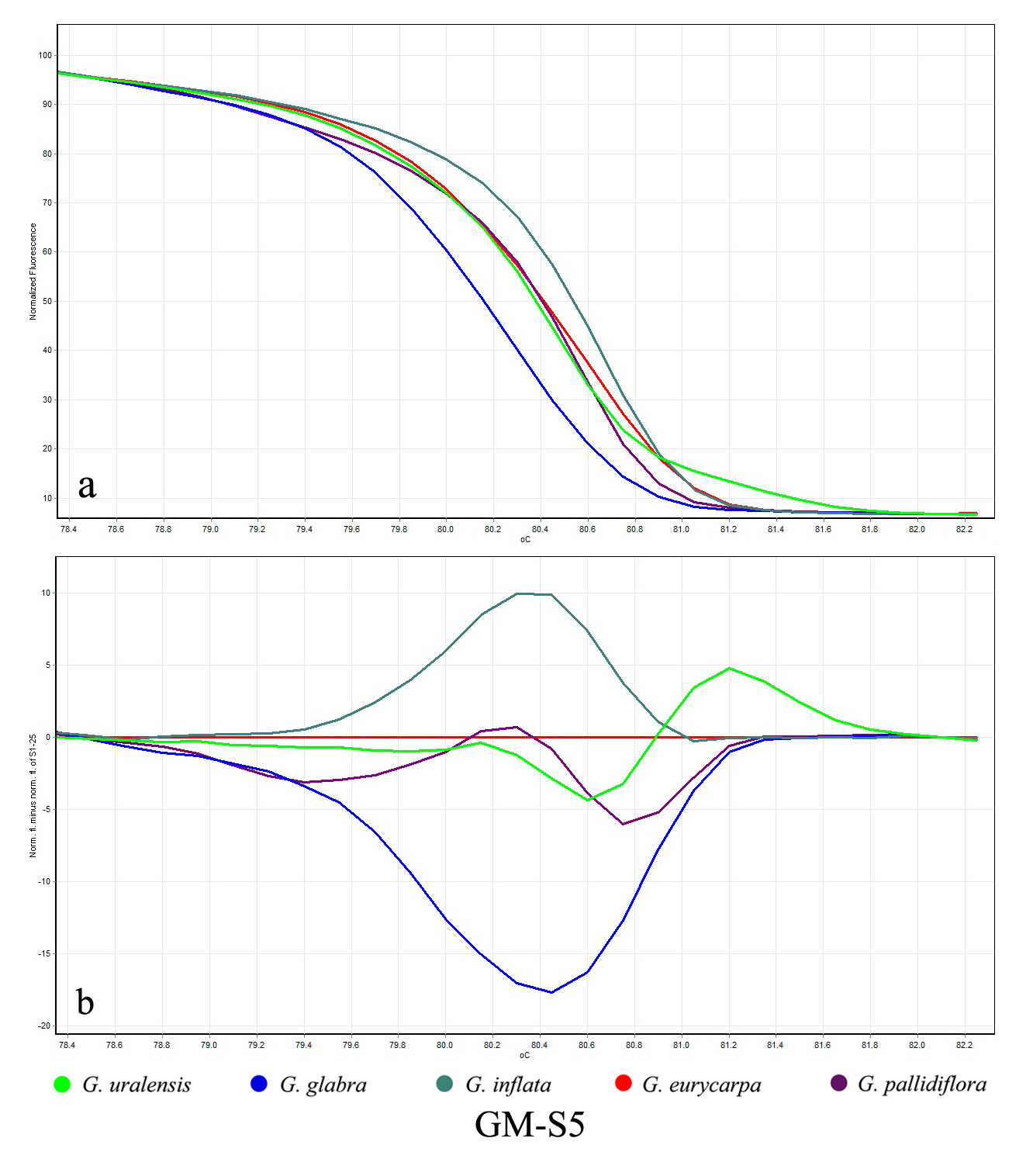

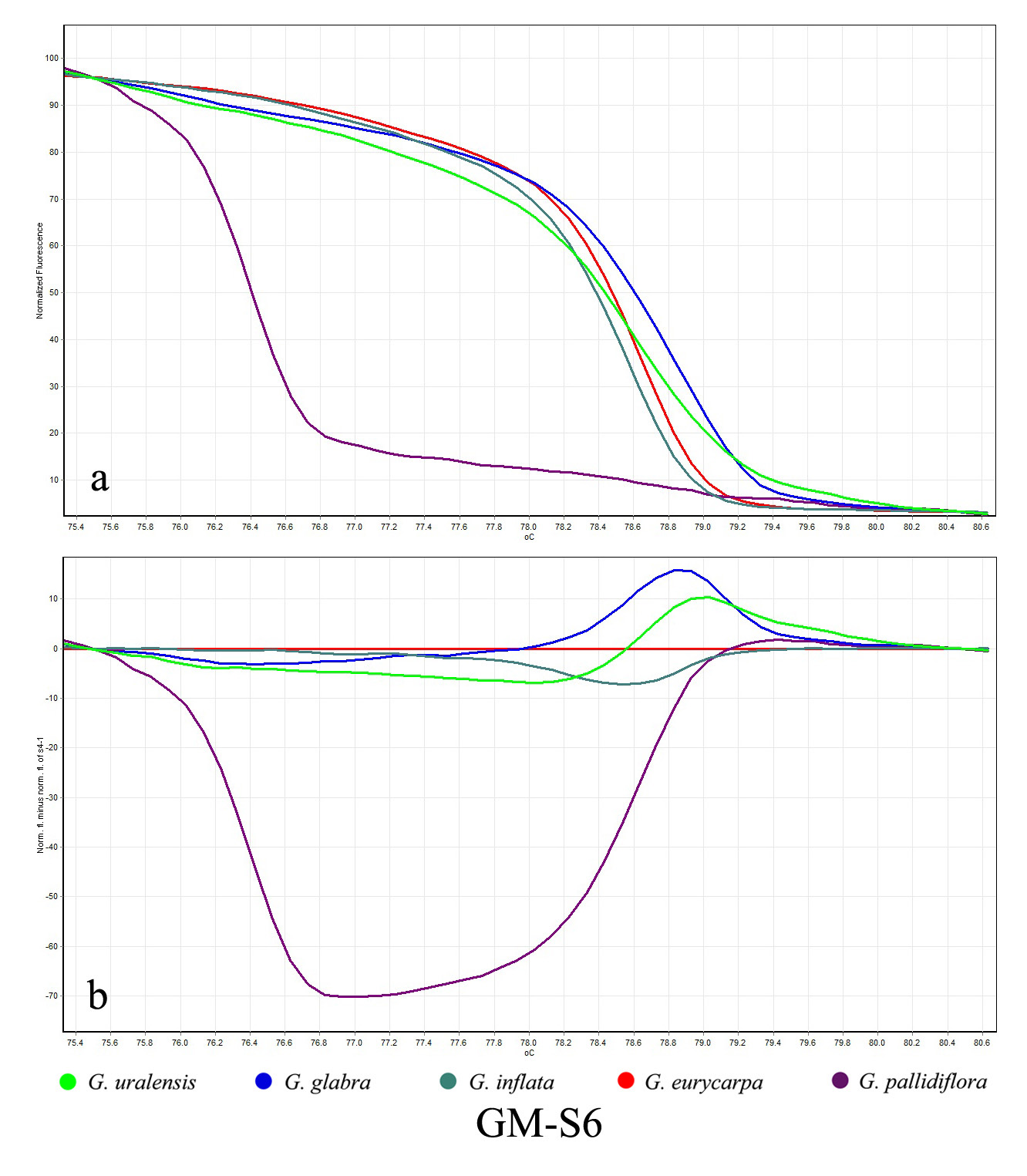

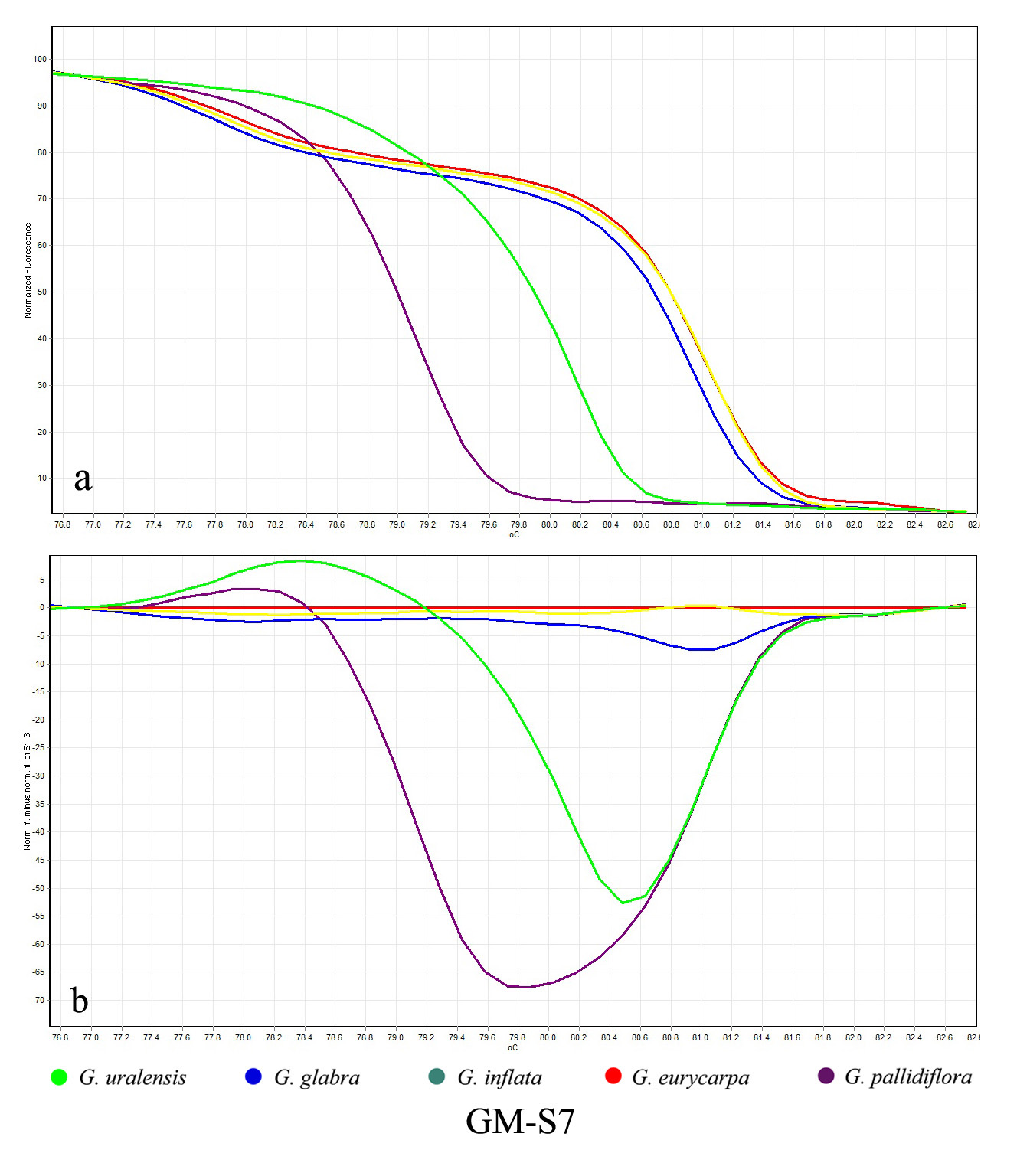

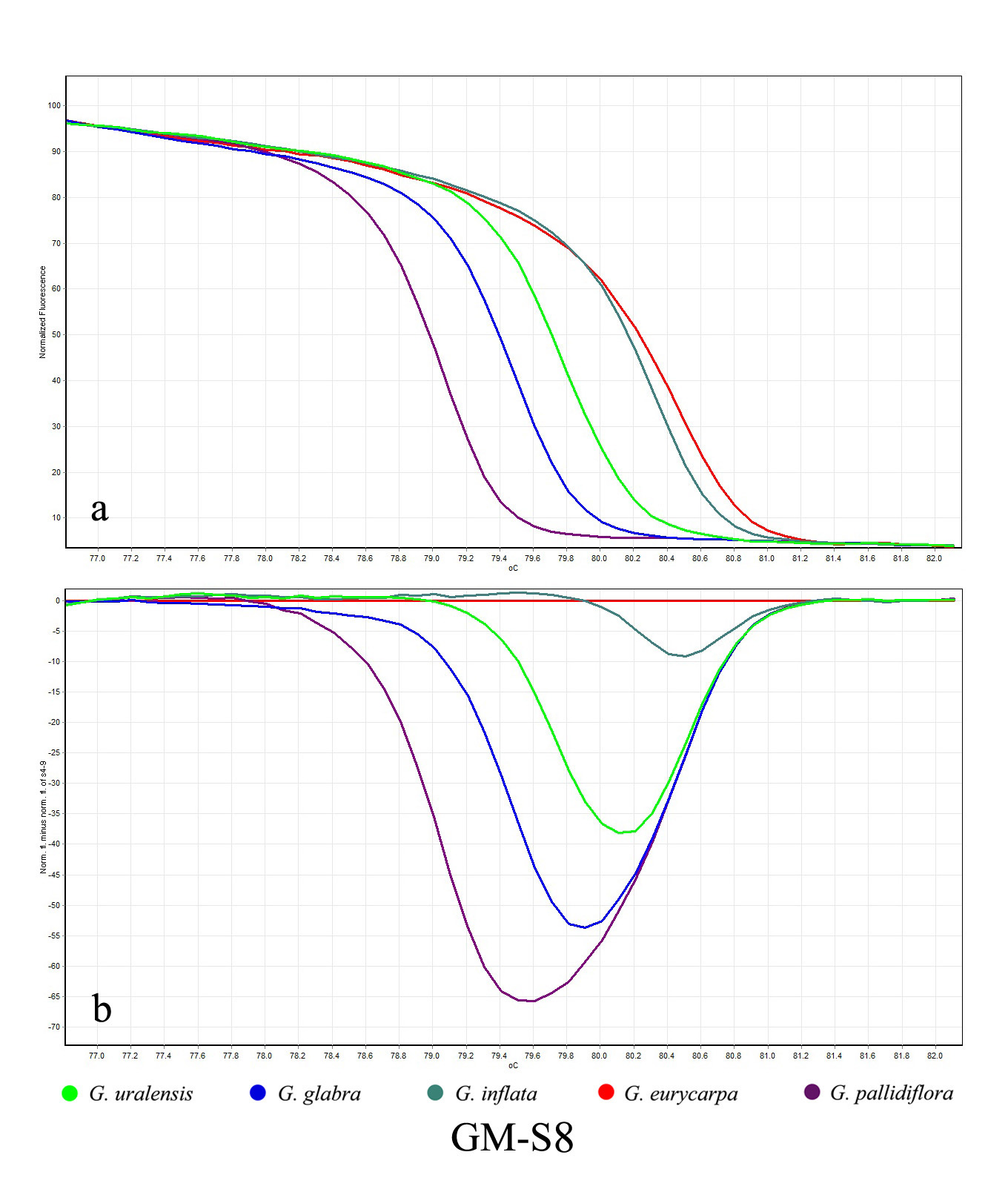


**Fig. S1.** Microsatellite typing of five *Glycyrrhiza* species using HRM analysis.


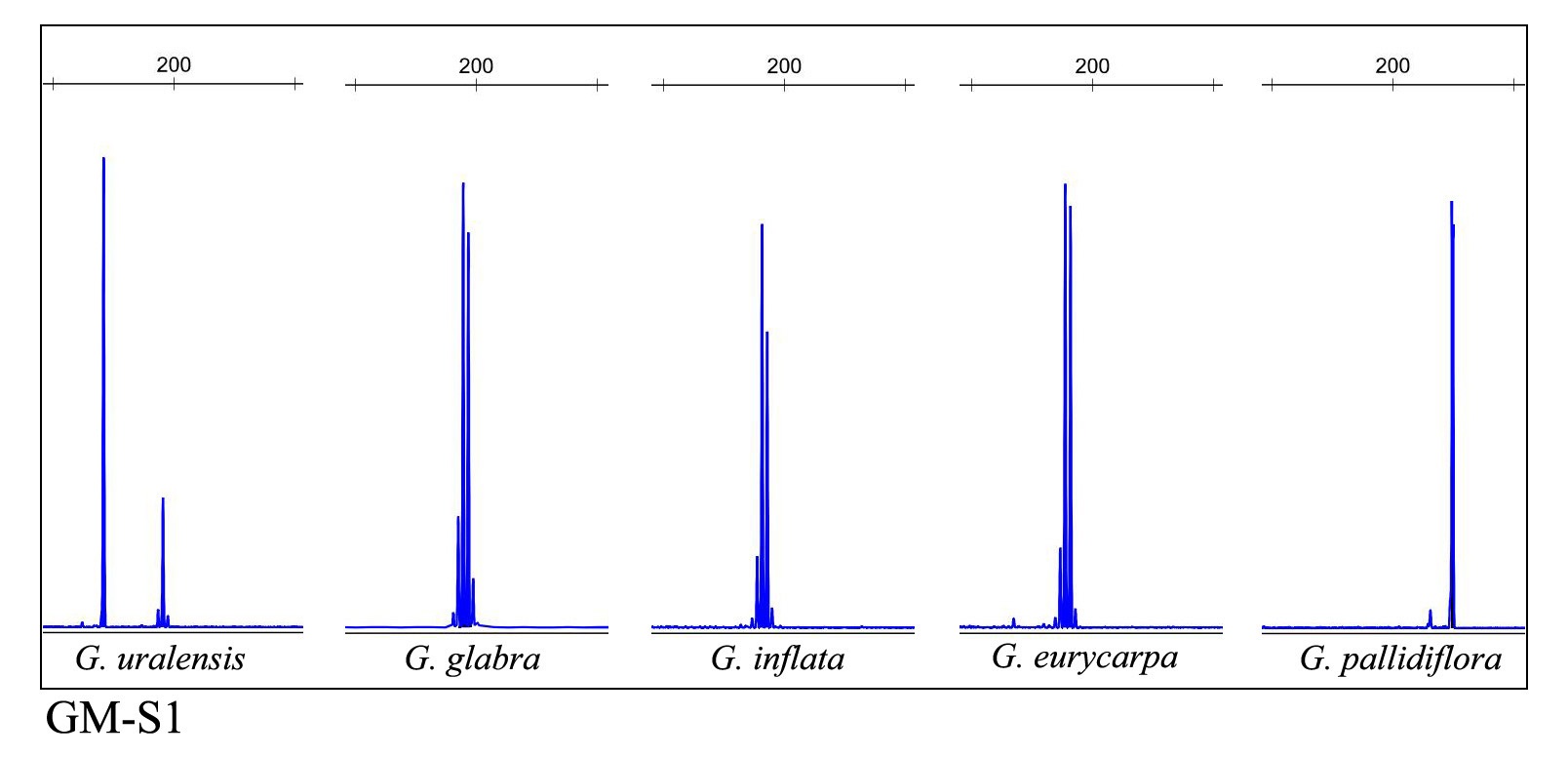

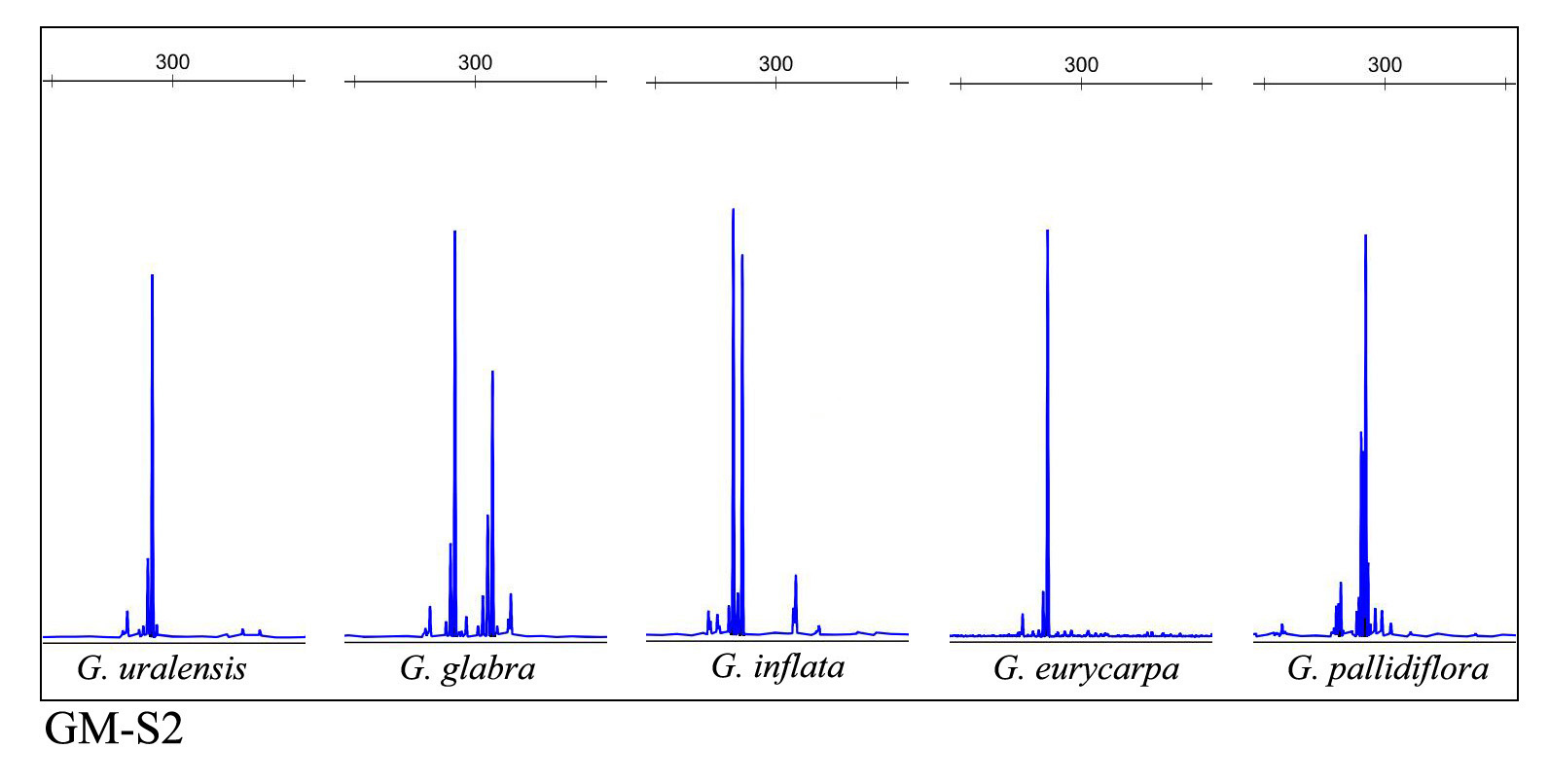

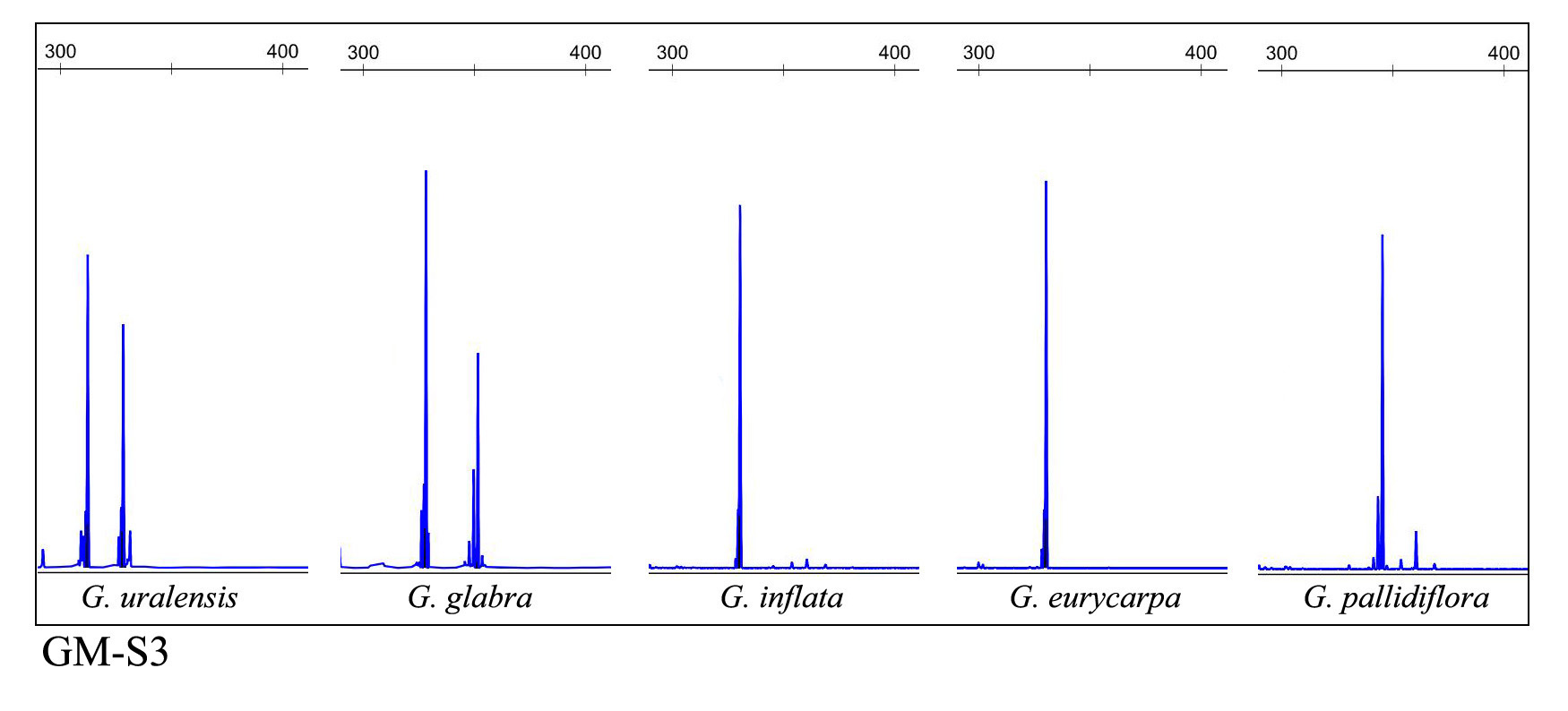

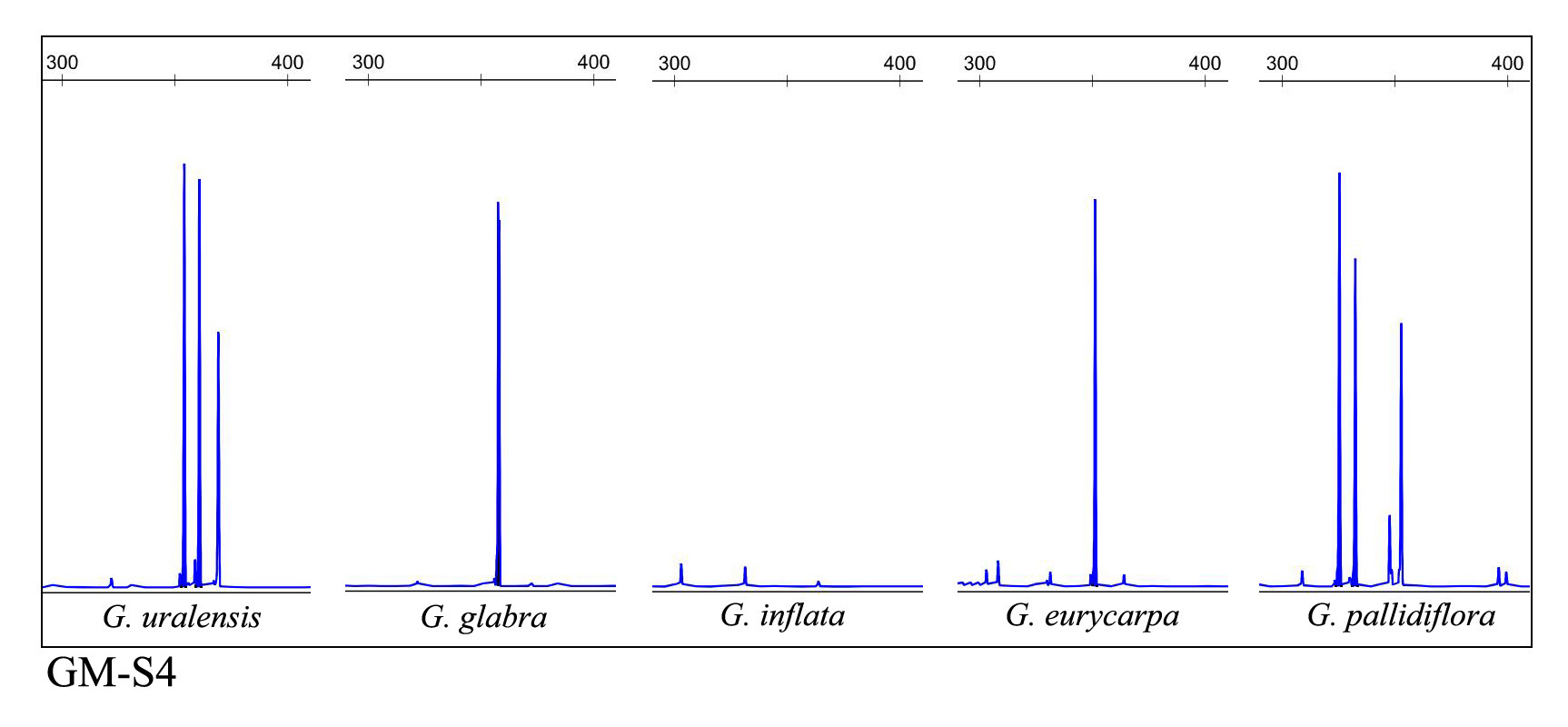

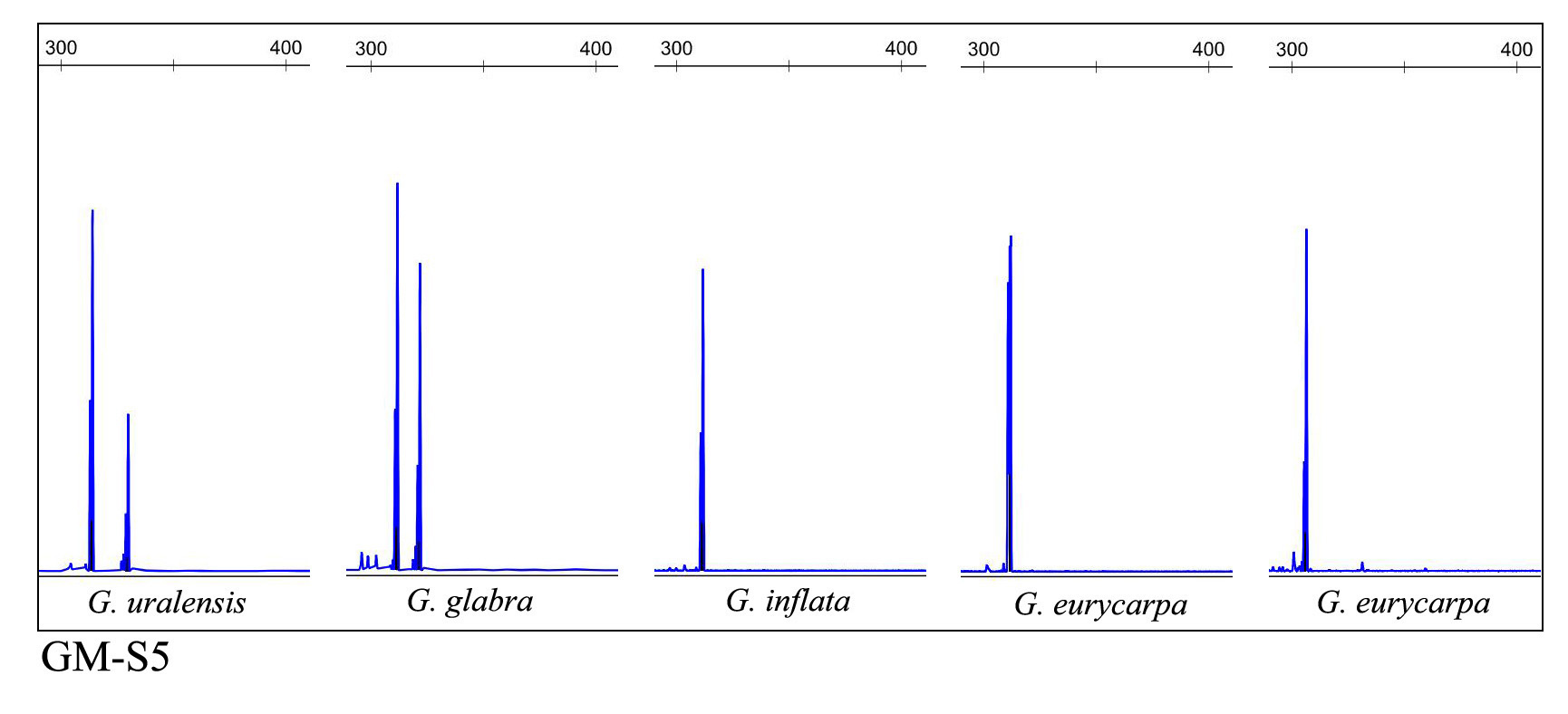

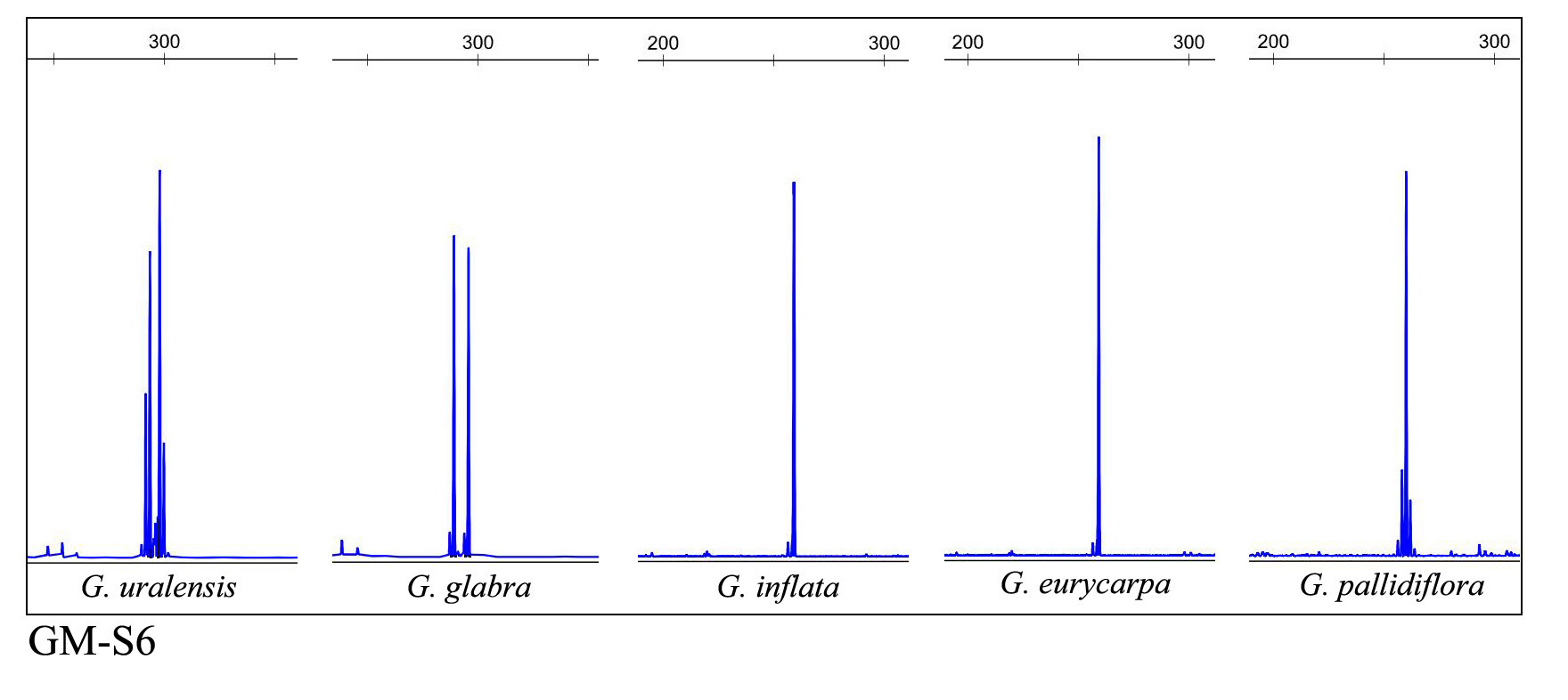

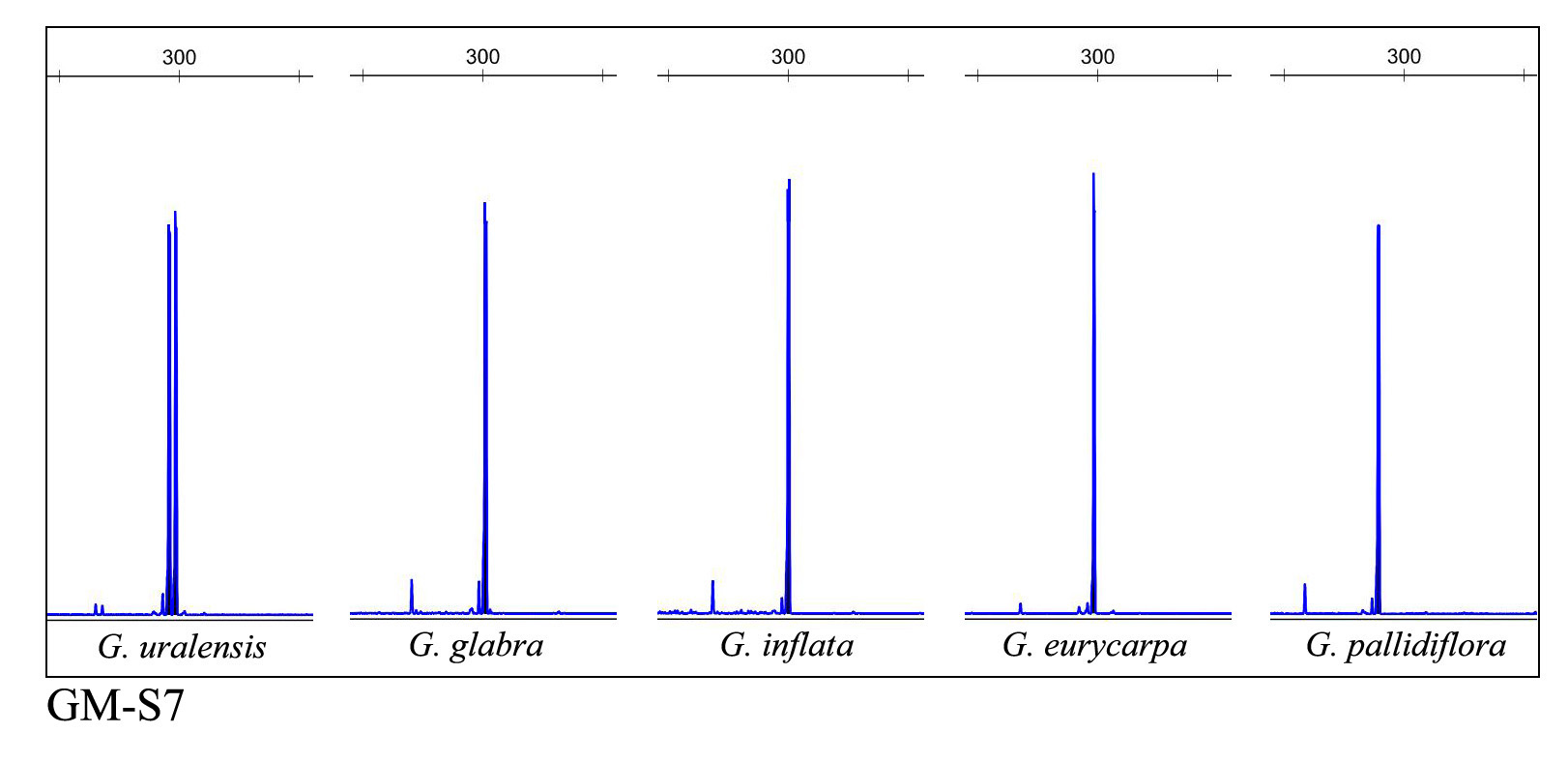

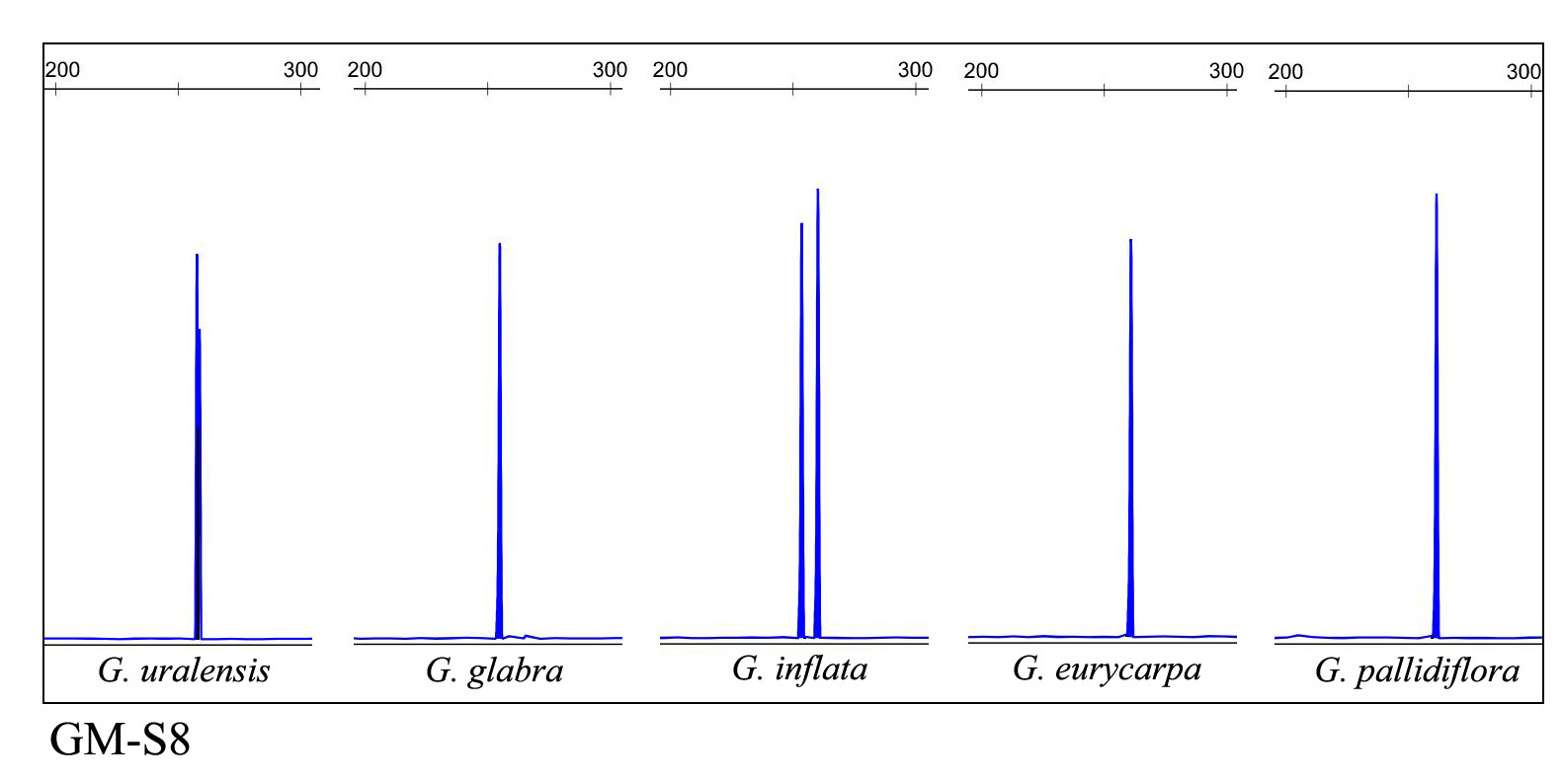


**Fig. S2.** Capillary electrophoresis profiles of the genotypes analyzed with the newly designed SSR markers.
